# Abundance of mitochondrial superoxide dismutase is a negative predictive biomarker for endometriosis-associated ovarian cancers

**DOI:** 10.1186/s12957-019-1565-0

**Published:** 2019-01-30

**Authors:** Tsukuru Amano, Tokuhiro Chano, Takahiro Isono, Fuminori Kimura, Ryoji Kushima, Takashi Murakami

**Affiliations:** 1 Department of Obstetrics and Gynecology, Shiga University of Medical Science, SetaTsukinowa-cho, Otsu, Shiga 520-2192 Japan; 2 Department of Clinical Laboratory Medicine, Shiga University of Medical Science, SetaTsukinowa-cho, Otsu, Shiga 520-2192 Japan; 3 Central Research Laboratory, Shiga University of Medical Science, SetaTsukinowa-cho, Otsu, Shiga 520-2192 Japan

**Keywords:** Clear cell ovarian carcinoma, Endometrioid ovarian carcinoma, Endometriosis-associated ovarian cancer, Mitochondrial superoxide dismutase

## Abstract

**Background:**

Endometrioid ovarian carcinoma and clear cell ovarian carcinoma are both classified as endometriosis-associated ovarian cancers (EAOCs). Despite the high rates of recurrence and mortality of EAOC, only a few prognostic biomarkers have been reported. Mitochondrial superoxide dismutase (SOD2) plays an important role in maintaining mitochondrial function through oxidative stress tolerance and contributes to chemotherapeutic resistance.

**Methods:**

To clarify the clinical significance of SOD2 in EAOC, SOD2 expression was semi-quantitatively investigated by immunohistochemical analysis in 61 primary EAOC cases, and the correlations between SOD2 expression and clinicopathological data and survival were analyzed.

**Results:**

Forty-six (75%) cases expressed high levels of SOD2. High SOD2 expression was associated with a poor prognosis on both univariate and multivariate analyses after adjusting for variables such as age, International Federation of Gynecology and Obstetrics (FIGO) stage, blood markers, histological type, and completion of treatment. There were 14 fatalities from 15 recurrences among 46 cases with high SOD2 expression. In contrast, only one recurrence and no fatalities were seen among 15 cases with low SOD2 expression.

**Conclusion:**

Increased SOD2 expression is a predictive biomarker for worse prognosis in EAOC. The therapeutic efficacy of the current standard therapeutic protocol for EAOC is limited; thus, mitochondrial SOD2 should be a therapeutic target for SOD2-abundant EAOC.

## Background

The incidence of ovarian cancer cases has been increasing, and the current rate is fourfold higher than the rate 40 years ago in Japan [1]. Endometrioid ovarian carcinomas and clear cell ovarian carcinomas account for 16.7% and 23%, respectively, of all ovarian cancer cases in Japan, and they are proportionally much more common than with ovarian carcinoma subtypes in Europe and the USA [2, 3]. Endometriosis-associated ovarian cancer (EAOC) usually occurs in younger women, with high recurrence and mortality rates [4]. In the clinical and pathological fields of EAOC, alterations in mismatch repair proteins and polymerase ε likely identified a subset of EAOCs with excellent outcomes, and abnormal p53 levels were related to a worse prognosis of EAOC [5]. Hormone receptor expression was associated with longer survival with EAOC [6], and glypican-3 was related to a worse prognosis of ovarian clear cell carcinomas [7]. However, further biomarkers predicting the prognosis are essentially required.

Inflammation and oxidative stress induced by heme and iron seem to be important triggers in the malignant transformation of endometriosis into EAOC [8]. EAOC is regarded as a stress-tolerant cancer because it arises from chocolate cysts with a highly oxidative microenvironment, induced by excess heme and chronic inflammation. In clear cell ovarian carcinoma, abnormalities are often found in genes responding to oxidative stress and metabolism of reactive oxygen species (ROS) [9].

Mitochondrial superoxide dismutase (SOD2) is an enzyme that metabolizes superoxide in mitochondria and plays an important role in maintaining mitochondrial function through oxidative stress tolerance. SOD2 overexpression correlates with poor prognosis in several cancers [10–12]. Suppression of SOD2 enhances ROS production in ovarian cancer cells and results in increased apoptosis, inhibition of proliferation, and enhanced sensitivity to chemotherapy [13]. However, in ovarian cancer, especially in EAOC, the correlation between SOD2 expression and prognosis remains unknown. It is unclear whether overexpression of SOD2 is strongly involved in ROS resistance and related to prognosis in EAOC. In the present study, we investigated the correlation between SOD2 expression and prognosis in a cohort of EAOC cases based on immunohistochemical staining of SOD2 and compared the results with clinicopathological data.

## Methods

### Patients

Sixty-one patients with EAOC treated at the Department of Obstetrics and Gynecology, Shiga University of Medical Science Hospital between 1998 and 2017 were enrolled in this study. The median patient age at the time of surgery was 48 (range, 30–80) years. The median follow-up periods were 66 (range, 1–156) months for progression-free survival (PFS) and 66 (range, 5–156) months for overall survival (OS). The endpoint of the observation period was defined as recurrence or progression by the last visit to the hospital for PFS and as death by the last visit to the hospital for OS. All patients initially underwent surgery and were categorically staged according to the International Federation of Gynecology and Obstetrics (FIGO) 2014 criteria. Of 61 patients, eight did not receive platinum-based adjuvant chemotherapy because of patient compliance or complications. The remaining 53 patients completed standard treatment with platinum-based chemotherapy following maximal surgical debulking. Therefore, all patients were chemotherapy-naive at the time of surgery. Pathological types of EAOC were 41 clear cell and 20 endometrioid carcinomas. Patient samples were selected from primary surgery specimens, including normal ovarian stroma, at the Shiga University of Medical Science Hospital. Forty-three cases (70.5%) had accompanying pathologically (36 cases) or clinically (7 cases) obvious endometriosis. Clinical and pathological data, age at diagnosis, FIGO stage, elevation in blood marker levels (serum carbohydrate antigen 19-9, serum carbohydrate antigen 125, or serum carcinoembryonic antigen), histological type, completion of treatment, and follow-up data were obtained from medical records. All included patients provided written informed consent. This study was carried out in compliance with the Declaration of Helsinki and was approved by the Shiga University of Medical Science Research Ethics Committee (reference number: 29-178).

## Immunohistochemistry

Formalin-fixed paraffin-embedded tissue was used for SOD2 immunohistochemical analysis. Samples were sliced into 5-μm sections. After dewaxing, the sections were autoclaved at 120 °C for 1 min in 10 mM sodium citrate buffer (pH 6.0) and immersed in 0.3% H_2_O_2_. They were then incubated overnight at 4 °C with primary antibodies against SOD2 (diluted 1:1000, #06-984, Millipore Corporation, Billerica, MA). Sections were rinsed with phosphate-buffered saline and incubated with secondary antibody conjugated with horseradish peroxidase (Simple Stain MAX-PO, Nichirei, Tokyo, Japan) at room temperature for 1 h. Sections were then stained with 3.3′-diaminobenzidine tetrahydrochloride and counter-stained with hematoxylin. All slides were scored by two pathologists blinded to the clinical data, using a standard light microscope. SOD2 expression in each EAOC was evaluated by relative comparison to the SOD2 expression of normal ovarian tissue stromal cells on the same slide. We categorized tumors into two groups on the basis of the intensity of SOD2 expression: (1) the high SOD2 group, wherein the intensity of the tumor cell staining was stronger than that of normal ovarian stromal cells, and (2) the low SOD2 group, wherein the intensity of the tumor cell staining was less or similar to normal ovarian stromal cells. As the S/N ratio of immunohistochemical SOD2 expression was quite high, the scoring of a case was inconsistent between the two pathologists, and the kappa statistic of SOD2 evaluation was 0.9567682. In one case with inconsistent scoring, obviously high SOD2 cell levels accounted for about 10% of tumor cells. We considered that such a population had a high probability of becoming seeds of recurrence or metastases; therefore, this case was eventually classified into the high SOD2 group.

### Statistical analysis

The Kaplan-Meier analysis for PFS and OS were performed using GraphPad PRISM 7 software (GraphPad Software Inc., La Jolla, CA, USA). All other statistical analyses were performed with SPSS statistical analysis software (version 22.0; SPSS Inc., Chicago, IL, USA).

Correlations between SOD2 expression and clinicopathological characteristics of the patients were evaluated using the chi-square test or Fisher’s exact test. OS and PFS were investigated using the Kaplan-Meier method. Survival curves were compared using log-rank and chi-square tests. Prognostic factors for PFS were evaluated by univariate and multi-variate analyses using Cox proportional hazards regression models. PFS intervals were used as an indicator for the hazard ratio (HR) and 95% confidence interval (CI). Significance was set at 0.05.

## Results

### Abundant SOD2 expression in EAOC and the correlation with clinicopathological characteristics

**Fig. 1:**
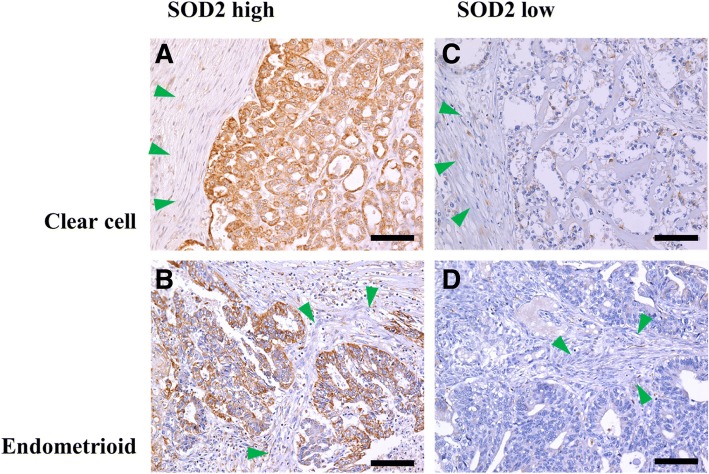
Examples of mitochondrial superoxide dismutase (SOD2) expression in endometriosis-associated ovarian cancers on immunohistochemical analysis. Cases subjected to immunohistochemical analysis for detection of mitochondrial superoxide dismutase (SOD2) expression in clear cell carcinoma and endometrioid carcinoma are shown in (**a**, **c**) and (**b**, **d**), respectively. High and low mitochondrial superoxide dismutase (SOD2) expression levels are demonstrated in (**a**, **b**) and (**c**, **d**), respectively. Green arrowheads indicate stromal cell areas, where SOD2 was subtly expressed and used as internal controls. The scale bars correspond to 100 μm

SOD2 expression was semi-quantitatively evaluated in 61 primary tumors of EAOC. Figure 1a–d show examples of mitochondrial superoxide dismutase (SOD2) expression in endometriosis-associated ovarian cancers on immunohistochemical analysis. In both endometrioid and clear cell carcinomas, SOD2 positivity was seen as strong dot-like structures in the cytoplasm, suggesting mitochondrial expression. SOD2 reactivity of normal ovarian stromal cells was used as an internal control of each histological section. Cases with stronger SOD2 staining of the tumor cells than normal ovarian stromal cells were categorized as high SOD2 cases. Among 61 tumors, 46 (75%) tumors expressed high levels of SOD2.

SOD2 expression was not affected by patients’ age, high FIGO tumor stage (stage 1–2 vs 3–4), presence of significant blood markers (yes vs no), histology (clear cell carcinoma vs endometrioid carcinoma), and optimality of adjuvant primary treatment (yes vs no) (Table 1).

**Table 1:** Correlation between mitochondrial superoxide dismutase (SOD2) expression and clinicopathological characteristics in 61 endometriosis-associated ovarian cancers

Characteristic	Cases, *n*	SOD2 expression, *n*		*P* value
		High (*n* = 46)	Low (*n* = 15)	
Age, years				
60<	11	10	1	0.2646
≤ 60	50	36	14	
FIGO class				
Low (I, II)	51	38	13	0.9999
High (III, IV)	10	8	2	
Blood marker				
Positive	48	37	11	0.7175
Negative	13	9	4	
Histology				
Clear cell	41	33	8	0.2164
Endometrioid	20	13	7	
Treatment				
Complete	53	41	12	0.3934
Incomplete	8	5	3	

Patients were stratified according to mitochondrial superoxide dismutase (SOD2) expression. SOD2 expression was not associated with age, International Federation of Gynecology and Obstetrics (FIGO) stage, blood markers, histology, or completeness of adjuvant primary treatment

### Abundant SOD2 expression can predict poor prognosis in EAOC

**Fig. 2:**
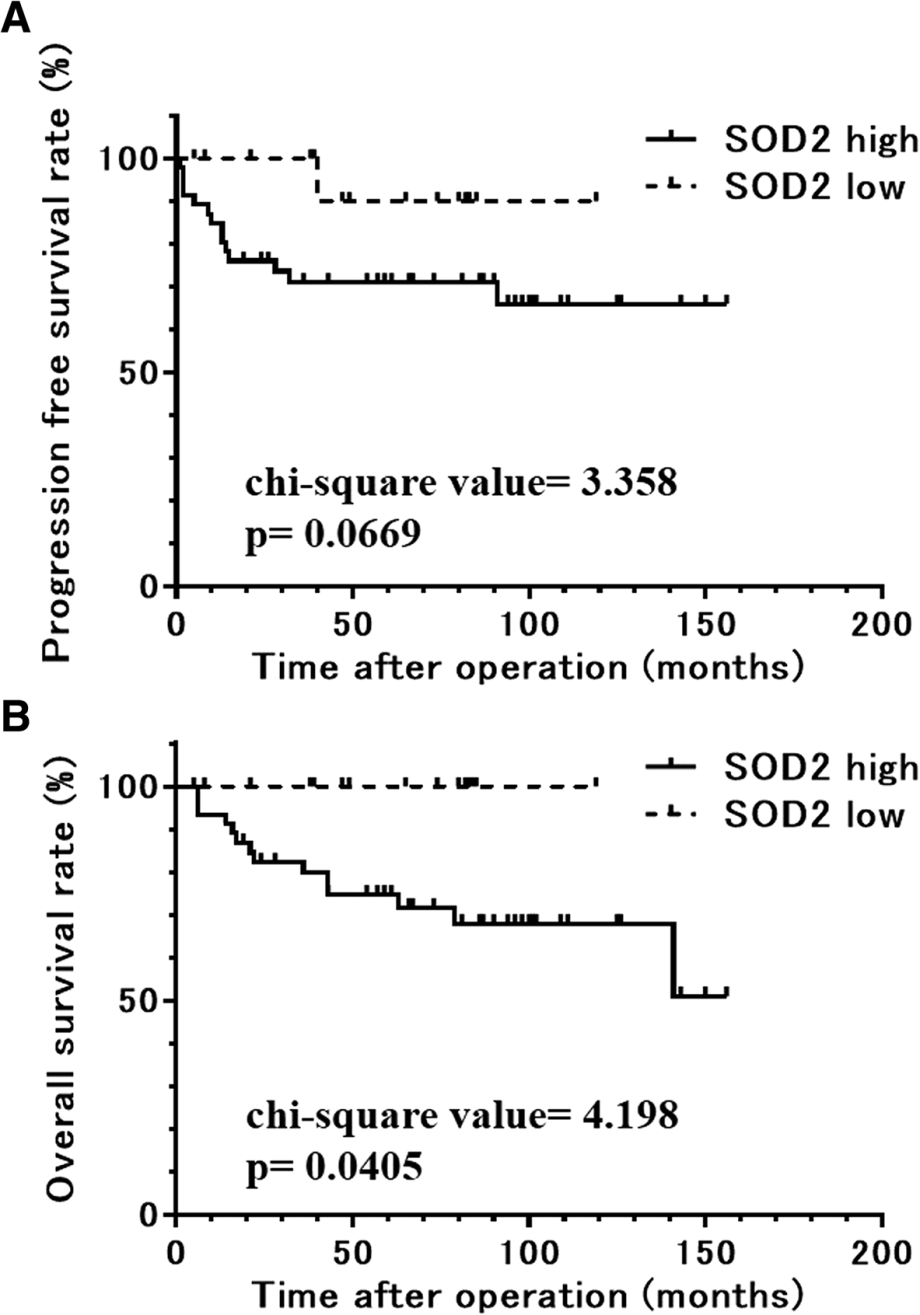
Progression-free (**a**) and overall (**b**) survival rates of the groups categorized by mitochondrial superoxide dismutase (SOD2) expression in a cohort of patients with endometriosis-associated ovarian cancer. A high expression of mitochondrial superoxide dismutase (SOD2) was associated with a trend toward a shorter progression-free survival (*χ*^2^ = 3.358, *p* = 0.0669) and was correlated significantly with poor overall survival (*χ*^2^ = 4.198, *p* = 0.0405) in endometriosis-associated ovarian cancers

Kaplan-Meier analysis demonstrated the association between high SOD2 expression and a trend toward shorter PFS (chi-square value = 3.358, *p* = 0.0669) and a significant correlation with poor OS (chi-square value = 4.198, *p* = 0.0405) in EAOC. The 5-year PFS rates in high and low SOD2 groups were 68.5% and 74.7% and those of OS in high and low SOD2 groups were 90.0% and 100%, respectively (Fig. 2). There were 14 fatalities from 15 recurrences among 46 cases in the high SOD2 group. In contrast, only one recurrence and no fatalities occurred among 15 cases in the low SOD2 group, and the single case of recurrence was completely rescued by chemotherapy following salvage surgery.

As cases with low SOD2 levels had notably better prognoses without any fatalities, it was impossible to perform Cox regression analysis for OS outcomes. In this case, we had to apply only PFS evaluation into the Cox proportional hazards model. On univariate analysis, high SOD2 expression, incomplete standard treatment, and high FIGO stage were found to be associated with poor prognosis. In addition, multivariate Cox proportional hazards regression analysis confirmed that high SOD2 expression was a significant independent predictive factor for poor PFS (HR = 8.929, 95% CI = 1.151–71.429, *p* = 0.0362; Table 2), as were incomplete standard treatment and high FIGO stage.

**Table 2:** Assessment of clinicopathological parameters predicting progression-free survival on univariate and multivariate analyses

			PFS univariate		PFS multivariate	
		*n*	HR (95% CI)	*p* value	HR (95% CI)	*p* value
Age	60</≤60	11/50	0.4890 (0.105–2.289)	0.3632		
FIGO class	III, IV/I, II	10/51	30.303 (7.353–125)	< 0.0001	19.608 (5.952–66.667)	< 0.0001
Blood marker	Positive/negative	48/13	0.806 (0.204–3.185)	0.7583		
Histology	Clear cell/endometrioid	41/20	2.212 (0.5778–8.475)	0.2463		
Standard treatment	Incomplete/complete	8/53	6.410 (1.488–27.778)	0.0126	7.576 (1.828–31.25)	0.0052
SOD2 expression	High/low	46/15	17.543 (1.825–166.667)	0.0130	10 (1.271–76.923)	0.0287

On univariate analysis, high expression of mitochondrial superoxide dismutase (SOD2), incompleteness of standard treatment, and high International Federation of Gynecology and Obstetrics (FIGO) stage were associated with poor prognosis. Multivariate Cox proportional hazards analysis also confirmed that high SOD2 expression, incompleteness of standard treatment, and high FIGO stage were significant prognostic factors for worse progression-free survival (PFS). *CI* confidence interval, *HR* hazards ratio

## Discussion

In this study, we identified two clinically important issues. First, the high SOD2 expression of cancer cells is a poor prognostic factor for EAOC. Second, the current standard therapeutic protocol is insufficient to achieve improved survival of patients with EAOC showing high SOD2 expression.

High SOD2 expression was a worse prognostic factor for EAOC. SOD2 plays an important role in ROS removal, whose production is induced by chemotherapeutic treatments, and maintenance of mitochondrial function. In previous studies, high SOD2 expression is associated with poor prognosis in some carcinomas [10–12]. Especially in renal clear cell carcinomas, which show pathological similarities with clear cell ovarian carcinomas, high SOD2 expression reflects better mitochondrial function and ROS resistance, and increased SOD2 expression correlates with poor prognosis [14]. The cancer genome atlas (TCGA) database cannot suggest that SOD2 is a prognostic indicator in ovarian cancer. However, as the database strongly depends on the overall incidence of cancer types, the provisional and final analyses of ovarian cancers have primarily focused on serous ovarian adenocarcinomas, rather than on EAOC including clear cell and endometrioid ovarian carcinomas. As EAOC often arises from endometriosis, a tissue abundantly exposed to inflammatory ROS, it is thought to acquire resistance to oxidative stress. Hemachandra et al. [15] revealed that SOD2 is more strongly expressed in ovarian clear cell carcinoma than in other epithelial ovarian cancer subtypes and that SOD2 is a pro-tumorigenic or metastatic factor. Hemachandra’s study conformed to the findings of the present study.

In the present cohort, no patients died in the low SOD2 expression group, even among patients with high stage and recurrence. All three patients including those with tumors with low SOD2 expression, who did not complete chemotherapy, have not had a relapse until now. In addition, two patients with high FIGO class and tumors with low SOD2 expression have survived without any cancer relapses. Regarding tumors with low SOD2 expression that are highly sensitive to chemotherapeutic ROS increments, the current standard therapeutic protocol, i.e., platinum-based chemotherapy following surgical resection, is considered sufficient. Increased ROS levels improve the antineoplastic effect of platinum-based chemotherapy [16]. This fact suggests that platinum-based chemotherapeutic agents are effective agents against EAOC with low SOD2 expression. Conversely, among cases with high SOD2 expression, 15 of 46 cases relapsed and 14 deaths were observed. These results indicate that the current therapeutic protocol should be considered insufficient in tumors with high SOD2 expression. With some anti-cancer agents, such as cisplatin, ROS are involved in the antitumor effect [17, 18]. As SOD2 is an antioxidant enzyme that can prevent oxidative redox-mediated damage of mitochondrial proteins and preserve mitochondrial function, EAOC with high SOD2 expression likely have strong resistance to oxidative stress caused by cancer treatment such as chemotherapy. In our cohort, SOD2 evaluation was performed from the sample before each chemotherapeutic treatment. Therefore, SOD2 may reflect the intrinsic aggressive character of the tumor in addition to the predisposed resistance to chemotherapy. EAOC, especially ovarian clear cell carcinomas, is well recognized to have greater resistance to platinum-based chemotherapy, a more aggressive clinical course, and more malignant behavior than other types of ovarian cancers [19–21]. Ovarian clear cell carcinomas have poor prognosis because of resistance to current chemotherapy protocols, based on platinum and taxane [19].

This study confirmed the following findings: a novel therapeutic strategy for clear cell ovarian carcinomas and/or other EAOCs, in which SOD2 is strongly expressed, should be established. Metformin and other biguanides, which have been clinically used for the treatment of diabetes mellitus, can inhibit complex I of the mitochondrial electron transport chain and mitochondrial respiration [22]. In addition, they exert anticancer effects on patients with diabetes diagnosed with pancreatic cancer, colon cancer, and prostate cancer [23–27]. By targeting tumor mitochondrial function, drug repositioning with the use of biguanides such as metformin has been proposed for some types of cancers [28]. Isono et al. [14] demonstrated that biguanide could improve the therapeutic effect in clear cell carcinomas in the kidney, which show similar pathology to ovarian clear cell carcinomas. Replacement therapy or drug repositioning to target tumor cell mitochondria, as can be achieved with the use of biguanides, should be investigated for the treatment of EAOC with abundant SOD2 expression, which is likely to retain stronger mitochondrial function.

As this study could not include a huge number of cases, it was impossible to analyze whether SOD2 was significantly predictive of the prognosis, after dividing into each category of FIGO clinical stage or histological type. Further analyses with more cases and a larger cohort of EAOC may be desired. Such studies will provide more supportive evidences for SOD2 as a predictive biomarker for EAOC.

## Conclusions

This study demonstrated that SOD2 abundance is a predictive biomarker for the poor prognosis in EAOC and highlighted the insufficiency of the current therapeutic standard against these tumors. Mitochondrial SOD2 should be regarded as an important therapeutic target for EAOC.

## Abbreviations

EAOC: Endometriosis-associated ovarian cancer
FIGO: International Federation of Gynecology and Obstetrics
OS: Overall survival
PFS: Progression-free survival
ROS: Reactive oxygen species
SOD2: Mitochondrial superoxide dismutase
